# ELK1 has a dual activating and repressive role in human embryonic stem cells

**DOI:** 10.12688/wellcomeopenres.15091.2

**Published:** 2019-07-05

**Authors:** Ian Prise, Andrew D. Sharrocks

**Affiliations:** 1 Faculty of Biology, Medicine and Health, University of Manchester, Manchester, M13 9PT, UK; 2 Faculty of Biology, Medicine and Health, University of Manchester, Manchester, M13 9PT, UK

**Keywords:** ELK1, repression, embryonic stem cells

## Abstract

**Background:** The ERK MAPK pathway plays a pivotal role in regulating numerous cellular processes during normal development and in the adult but is often deregulated in disease scenarios. One of its key nuclear targets is the transcription factor ELK1, which has been shown to play an important role in controlling gene expression in human embryonic stem cells (hESCs). ELK1 is known to act as a transcriptional activator in response to ERK pathway activation but repressive roles have also been uncovered, including a putative interaction with the PRC2 complex.

**Methods:** Here we probe the activity of ELK1 in hESCs by using a combination of gene expression analysis in hESCs and during differentiation following ELK1 depletion and also analysis of chromatin occupancy of transcriptional regulators and histone mark deposition that accompany changes in gene expression.

**Results:** We find that ELK1 can exert its canonical activating activity downstream from the ERK pathway but also possesses additional repressive activities. Despite its co-binding to PRC2 occupied regions, we could not detect any ELK1-mediated repression at these regions. Instead, we find that ELK1 has a repressive role at a subset of co-occupied SRF binding regions.

**Conclusions:** ELK1 should therefore be viewed as a dichotomous transcriptional regulator that can act through SRF to generate both activating and repressing properties at different genomic loci.

## Introduction

*In vitro* studies on human embryonic stem cells are an important step in understanding the molecular basis to human development. Cultured human embryonic stem cells (hESCs) require FGF2-mediated signalling through the ERK pathway to maintain their pluripotent state ( Lanner & Rossant, 2010). More recent studies indicate that an earlier ERK pathway-independent state can be achieved whereby ERK pathway suppression is a key event in driving this transition ( Theunissen *et al*., 2014). This earlier state is equivalent to the mouse ESC naïve ground state that is thought to represent the pre-implantation epiblast. Nevertheless, understanding the role of the ERK pathway in hESCs remains an important goal. Some of the best characterised targets of the ERK MAPK signalling pathway are the E-twenty six (ETS) proteins, which are nuclear transcription factors and as such can directly convert ERK pathway signalling events into changes to the cellular transcriptome ( Yang *et al*., 2013). One of the best-studied ETS transcription factors in this context is ELK1, which is multi-phosphorylated by ERK in its transactivation domain, thereby converting it into a potent transcriptional activator ( Cruzalegui *et al*., 1999; Gille *et al*., 1995; Janknecht *et al*., 1993; Marais *et al*., 1993; Mylona *et al*., 2016). Recently, ERK was shown to exhibit a high degree of overlap with ELK1 binding to chromatin in hESCs, and this association was observed at active chromatin regions, consistent with an activating function for ELK1 ( Göke *et al*., 2013). However, unexpectedly, ELK1 was also found to bind to a different set of genomic loci, which were co-occupied with PRC2 complex components and marked with repressive histone tail modifications. This observation is suggestive of a repressive role in this context, and a model was proposed in which ELK1 promotes PRC2 complex recruitment and hence transcriptional repression. ELK1 has previously been associated with transcriptional repression through its ability to recruit the SIN3A complex in response to growth factor signalling ( Yang *et al*., 2001) and the recruitment of HDAC2 following its modification with SUMO ( Yang & Sharrocks, 2004). SIN3A complex recruitment is associated with inactivation of ELK1 following activation by ERK-mediated phosphorylation, whereas SUMO-mediated HDAC2 recruitment is thought to maintain ELK1 in a transcriptionally repressive state prior to growth factor stimulation. A further repressive mechanism has been associated with ELK1, whereby it competes for binding of SRF with the potent transcriptional co-activator myocardin and other MRTF family members ( Wang *et al*., 2004; Zaromytidou *et al*., 2006). More recently, a similar repressive role for ELK1 was observed, but instead of competing for co-activator binding to SRF, ELK1 competed with other activating transcription factors from the ETS family for directly binding to their recognition sites on DNA ( Odrowaz & Sharrocks, 2012). ELK1 therefore appears to be a bifunctional transcription factor that acts as an ERK-dependent activator through its binding partner SRF but also has numerous other repressive roles.

Here we extended the analysis of ELK1 function in hESCs, first exploring the relationship between ELK1 and the PRC2 complex, and then its activity through its known binding partner SRF. Despite ELK1 and PRC2 co-occupying a large number of genomic regions, we were unable to uncover evidence to support a repressive role of ELK1 in this context. However, unexpectedly, we were able to uncover a repressive role for ELK1 in the context of a subset of SRF-bound regulatory regions. This repressive role appeared distinct from a simple competition model for SRF binding by the co-activator MRTFA. ELK1 therefore possesses both activating and repressive functions in hESCs, directed through its regulatory partner protein SRF.

## Results

### Functional interplay between ELK1 and PRC2

**Figure 1.**
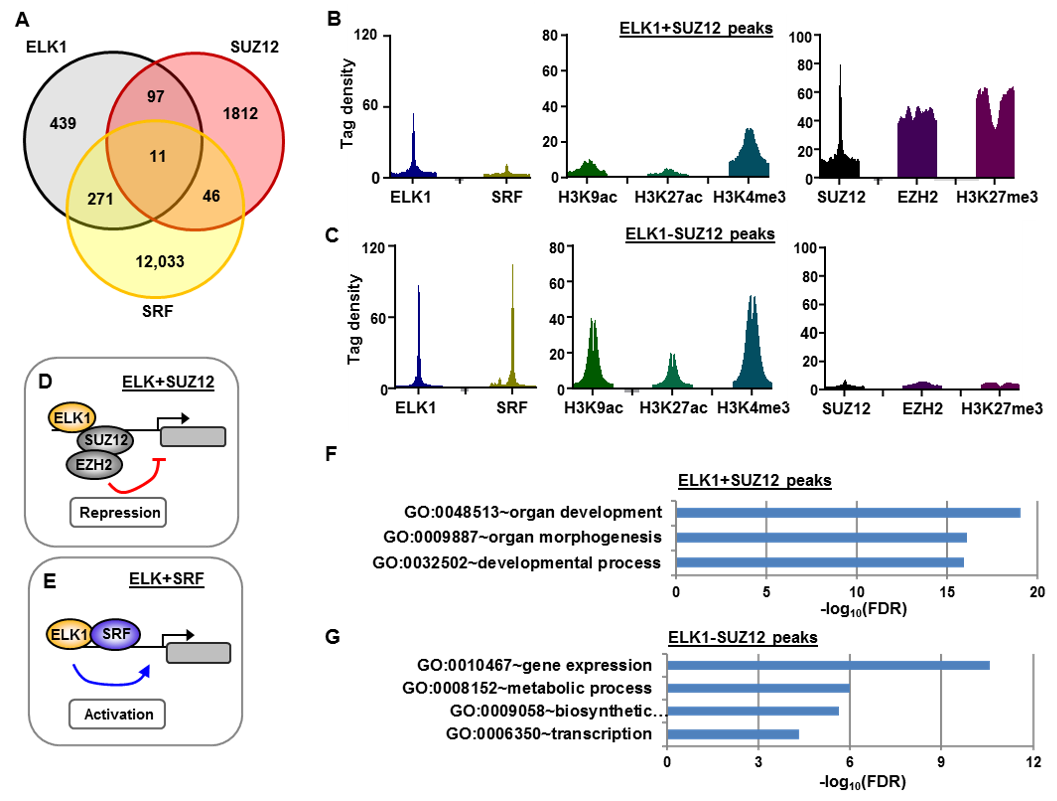
ELK1 has binding modules either enriched for PRC2 or enriched for active histone marks.

Previous studies demonstrated that ELK1 occupies two distinct sets of genomic loci in H1-hESCs ( Göke *et al*., 2013). One set was associated with co-binding with SRF, a configuration which is usually associated with transcriptional activation. However, the second set of loci exhibited co-localisation with members of the repressive PRC2 complex, hence suggesting a role in transcriptional repression. As these conclusions were based on the analysis of promoter-proximal ELK1 binding sites, we re-analysed the chromatin immunoprecipitation sequencing (ChIP-seq) data to establish whether these patterns could be observed in a genome-wide manner. Initially we focussed on the co-association with the PRC2 complex, and segregated ELK1 regions according to whether co-binding of the PRC2 complex subunit SUZ12 could be identified (ELK1+SUZ12) or not (ELK1-SUZ12). A significant overlap in ELK1 and SUZ12 binding regions was observed (108 regions; hypergeometric p-value = 7.4×10 ^-12^), although the majority of ELK1 binding peaks showed no overlap ( Figure 1A). Next, we asked whether the two classes of ELK1 binding regions showed differences in co-association with the ELK1 binding partner SRF and a variety of histone marks that are characteristic of transcriptional repression or activation. The ELK1+SUZ12 binding loci were enriched for SUZ12 and EZH2 binding and for the H3K27me3 histone modification, suggesting repressed regions of chromatin ( Figure 1B). These regions showed no enrichment of the ELK1 DNA binding motif as observed previously for a smaller window ( Göke *et al*., 2013), suggesting an indirect mechanism for recruitment of ELK1 to chromatin at these loci. In contrast, the ELK1-SUZ12 regions showed little co-association with the repressive features and instead high enrichment of SRF binding was observed and co-association with histone marks suggestive of active transcription, H3K9ac, K3K27ac and H3K4me3 ( Figure 1C). These “active” sites also showed strong occupancy by its known binding partner, SRF, consistent with a large overlap between ELK1 binding regions when analysed at the individual binding peak level ( Figure 1A). These data point to the existence of an active ELK1+SRF module that lacks PRC2 binding, and a distinct repressive ELK1+SUZ12 module ( Figure 1D, E) and are broadly consistent with the models previously proposed using a subset of this data ( Göke *et al*., 2013).

( **A**) Venn diagram showing the intersection of binding regions from genome-wide chromatin immunoprecipitation-sequencing datasets for ELK1, SUZ12 and SRF in H1-hESCs. ( **B** and **C**) Transcription factor and histone modification tag density profiles in H1-hESCs in a region 2,500 bp to either side of the centre of the ELK1 binding regions. ELK1 binding regions are portioned according to ( **A**) overlapping with SUZ12 binding regions (+SUZ12) or ( **B**) not overlapping with SUZ12 binding regions (-SUZ12). ( **D** and **E**) diagrammatic illustration of the ELK1 binding regions associated with co-binding of the repressive PRC2 complex ( **D**) or associated with its binding partner SRF and active regions of chromatin ( **E**). ( **F** and **G**) Biological function gene ontology terms of genes associated with ELK1 binding loci either overlapping with SUZ12 binding loci ( **D**) or not overlapping with SUZ12 binding loci ( **E**).

Next, we focussed on the potential repressive role of ELK1 in the context of the ELK1+SUZ12 co-bound regions and sought to establish whether the genes associated with these peaks had potential biological relevance. To address this, we first assigned genes to binding loci with HOMER ( Heinz *et al*., 2010), using the nearest TSS model. We next analysed the biological process gene ontologies (GO) of genes linked to partitioned ELK1 binding loci. ELK1 binding loci overlapping with SUZ12 binding loci (ELK1+SUZ12) were enriched for terms relating to development ( Figure 1F). Terms are provided on figshare ( Prise, 2019a). This result is consistent with the known function of PRCs in functionally repressing key developmental genes in hESCs ( Lee *et al*., 2006; Yu *et al*., 2017). However, an ELK1-mediated mechanism of PRC repression would be novel. These loci are also enriched for the H3K27me3 modification ( Figure 1B), a functional indication of PRC2-mediated repression, suggesting that an ELK+SUZ12 module might be involved developmental gene repression. In contrast, we found that ELK1-binding loci not overlapping with SUZ12 binding loci were not enriched for developmental processes and were instead correlated with gene expression and metabolic processes ( Figure 1G). These latter observations are consistent with the role for ELK1 previously identified in HeLa cells ( Boros *et al*., 2009). 

**Figure 2.**
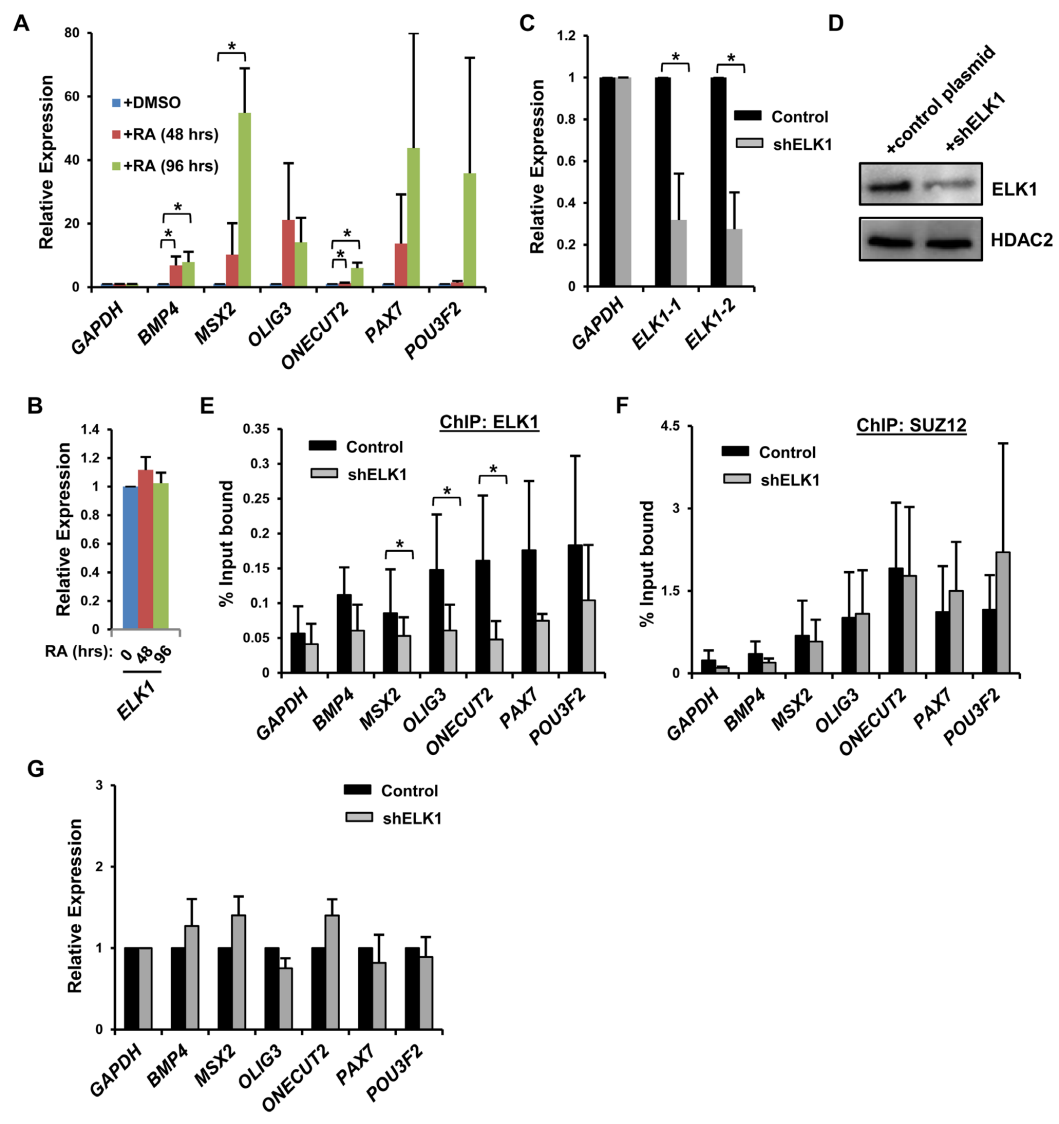
ELK1 is not involved in PRC2-mediated repression. ( **A** and **B**) Reverse transcription-quantitative PCR (RT-qPCR) analysis of ELK1+SUZ12-bound genes ( **A**) and *ELK1* ( **B**) upon 48 and 96 hours of retinoic acid (RA) treatment. Data are shown relative to DMSO-treated cells (taken as 1) and are the average of at least three independent experiments. Asterisks represent p-value <0.05. ( **C**) RT-qPCR of *GAPDH* and *ELK1* expression after 96 hours of shELK1 knockdown in H1-hESCs. Two different shRNA vectors were tested (plasmid containing shRNA_2 was subsequently used in all experiments). Data are shown relative to control empty plasmid treated cells (taken as 1) and are the average of three independent experiments. Asterisks represent p-value <0.05. ( **D**) Western blot analysis of ELK1 and HDAC1 expression in H1-hESC after 96 hours of treatment with an empty plasmid or a plasmid containing shELK1. ( **E** and **F**) Chromatin immunoprecipitation-qPCR of ELK1-binding regions for ELK1 ( **E**) or SUZ12 ( **F**) occupancy after 96 hours of treatment with either control empty vector or shELK1. ( **G**) RT-qPCR of ELK1+SUZ12-bound genes after 96 hours of treatment with either control empty vector or shELK1. Data are normalized to *GAPDH* expression and “control plasmid” (taken as “1”) and are the average of 5 independent experiments, with the exception of the data for *ONECUT2* and *POU3F2* which are the average of 4 independent experiments.

Having shown that ELK1+SUZ12 peaks were enriched in development genes, we then identified a set of associated target genes for further study that are induced upon differentiation. We hypothesised that the regulatory regions of these genes would switch from repressed (and PRC2-bound) to active during differentiation. We chose to use retinoic acid (RA), a potent initiator of hESC differentiation. Using the nearest TSS-association model in HOMER, we identified a set of ELK1+SUZ12-bound genes whose expression increased upon 48–96 hours of treatment with RA and focussed on six of these that showed robust induction ( Figure 2A). *ELK1* expression is unaffected under these conditions ( Figure 2B). To understand the role of ELK1 in the context of the repressive modules associated with these genes, we depleted ELK1 using shRNA ( Figure 2C, D) and examined the effect on PRC2 occupancy and gene expression in H1-hESCs. ELK1 depletion led to the expected decrease in ELK1 binding in these regions ( Figure 2E). However, this was not accompanied with a decrease in SUZ12 binding ( Figure 2F), nor did an ELK1 knockdown result in a substantive increase in nearby gene expression ( Figure 2G). Thus, although ELK1 co-occupies a set of genomic regions with the PRC2 complex, these results indicate that ELK1 does not appear to have a role in maintaining PRC2 occupancy or in maintaining transcriptional repression through these regions in H1-hESCs. Raw RT-qPCR ( Prise, 2019b) and ChIP-qPCR ( Prise, 2019c) data are available on figshare.

### Functional interplay between ELK1 and SRF

**Figure 3.**
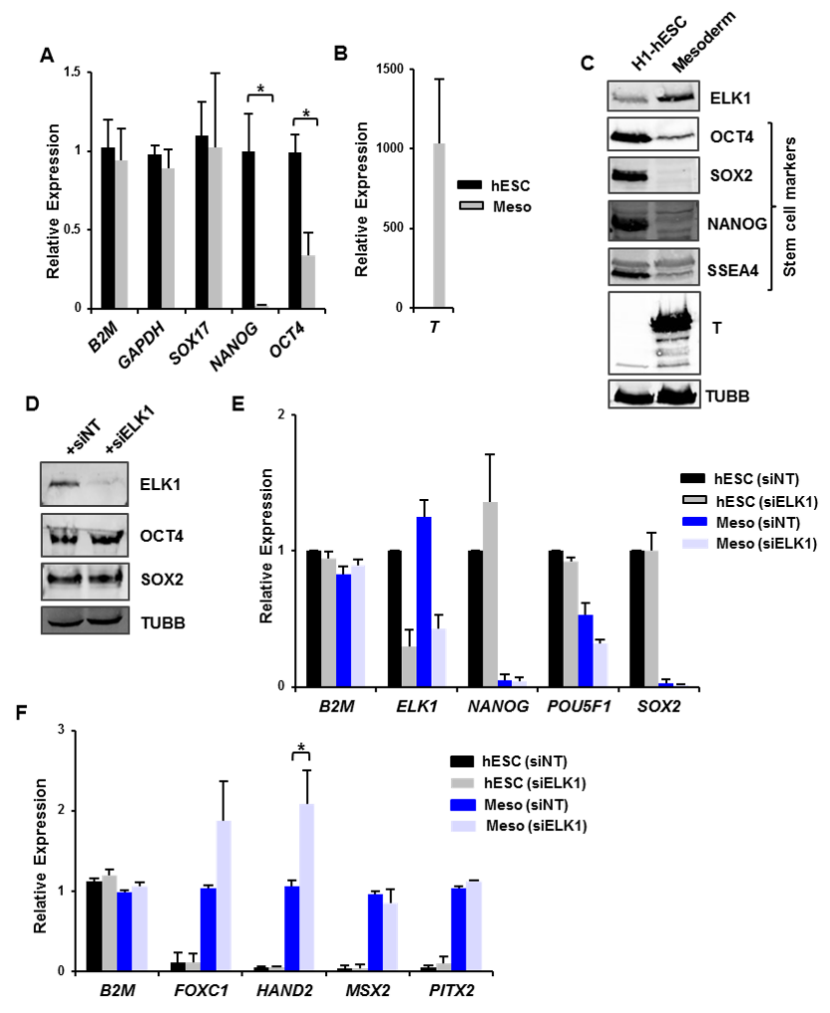
The role of ELK1 in gene expression during mesoderm differentiation. ( **A** and **B**) Reverse transcription-quantitative PCR (RT-qPCR) measuring the expression of pluripotency markers ( **A**) or the mesoderm marker ( *T/Brachyury*) after 72 of growth in MIM. ( **C**) Western blots for ELK1, the indicated pluripotency markers, T/Brachyury, and the control TUBB protein in H1-hESC cells and H1-hESCs grown in MIM for 72 hours (mesoderm cells). ( **D**) Western blots for ELK1, OCT4, SOX2, and TUB expression in H1-hESC cells treated with non-targeting (NT) siRNA or siRNAs targeting ELK1. ( **E** and **F**) RT-qPCR measuring the expression of ELK1 and the indicated pluripotency markers ( **E**) or the indicated ELK1-bound genes in H1-hESCs and H1-hESCs after 98 hours of growth in MIM (mesoderm cells) ( **F**) after treatment with non-targeting (NT) siRNA or siRNAs targeting ELK1. RT-qPCR data are normalised to H1-hESCs in the presence of siNT and are the average of 3 independent experiments. *p-value < 0.05.

Having shown that ELK1 plays no clear role in PRC2-mediated gene repression, we next returned to the ELK1 module, which preferentially shows enrichment for co-binding of SRF. Of the 710 ELK1-bound regions not found in the ELK1-SUZ12 dataset, 282 (38%) also show SRF co-occupancy ( Figure 1A; hypergeometric p-value= 3.2 × 10 ^-209^). Notably, there are very few regions co-bound by ELK1, SRF and SUZ12, indicating a clear distinction between regions bound by ELK1 and either SRF or SUZ12. We decided to focus on the role of ELK1 in differentiation to mesoderm as SRF has previously been implicated in this developmental process ( Arsenian *et al*., 1998). An additional advantage of the mesoderm differentiation protocol is that it produces a decrease in pluripotency factors and high expression of the marker gene *T* after a short 3-day treatment time ( Figure 3A–C), and is therefore compatible with a siRNA depletion approach. ELK1 knockdown revealed no general change in expression of pluripotency factors in H1-hESCs ( Figure 3D, E). ELK1 protein expression increased in mesoderm cells ( Figure 3C) but there was little change in the expression of two mesoderm-marker genes *MSX2* and *PITX2* following ELK1 depletion, indicating the absence of a general role in mesoderm differentiation ( Figure 3F). In contrast two other marker genes ( *FOXC1* and *HAND2*) exhibited increased expression following differentiation to mesoderm, suggesting a potential repressive function in this context ( Figure 3F). Only *FOXC1* is bound by ELK1 in hESCs, pointing to a potential direct role in this context and as ELK1 is co-bound with PRC2 components at this locus, it leaves open the possibility of a gene-specific rather than genome-wide role for this complex in mesoderm cells. Equally, ELK1 shows co-binding with SRF (see Figure 5 below) so may be repressing through that complex. It should however be noted that the increase in expression for *FOXC1* was not significant.

**Figure 4.**
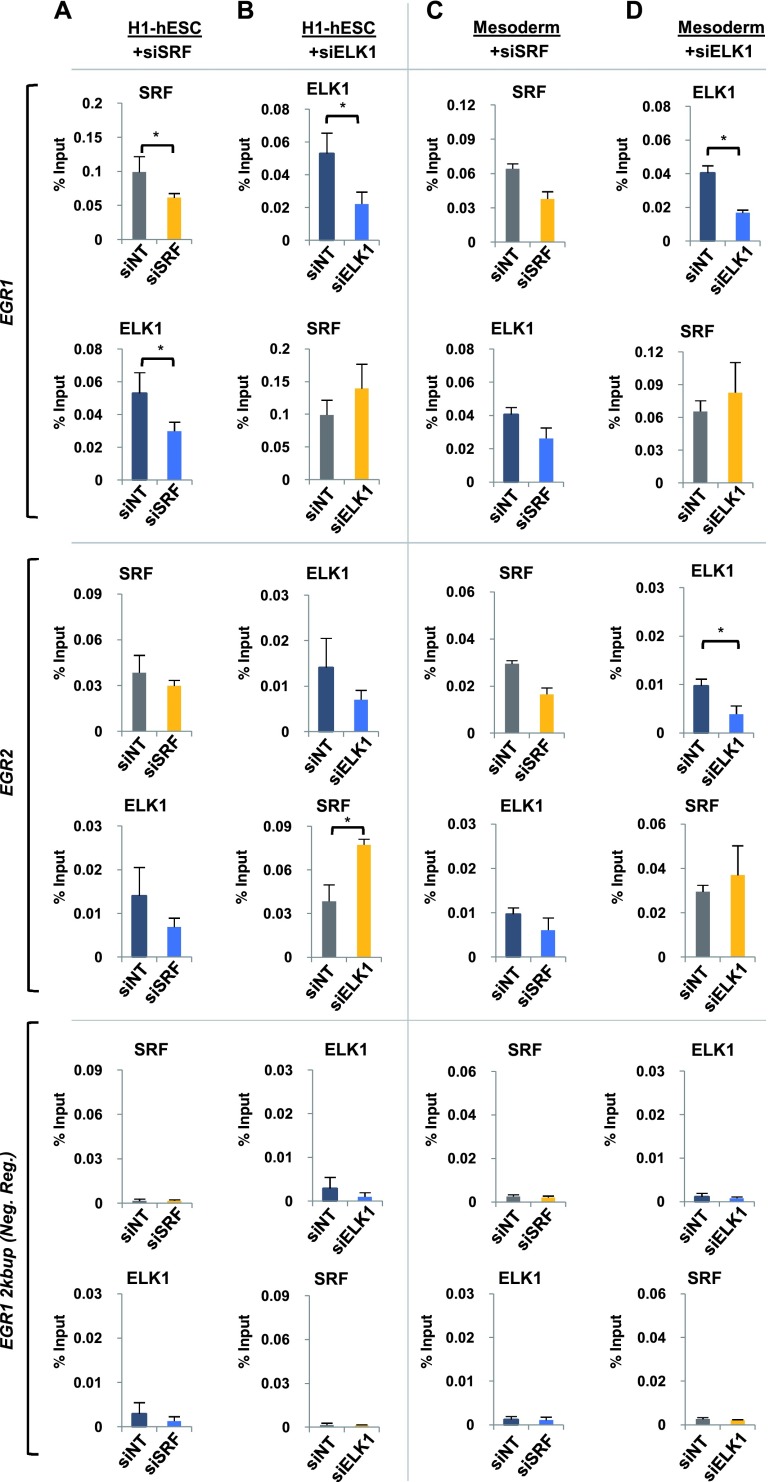
Reciprocal effects of ELK1 and SRF depletion on each other’s chromatin association. ( **A**– **D**) Chromatin immunoprecipitation-quantitative PCR showing ELK1 and SRF binding (indicated above each graph) after treatment of H1-hESCs ( **A** and **B**) or mesoderm cells ( **C** and **D**) with non-targeting (NT) siRNA or siRNAs against SRF ( **A** and **C**) or siELK1 ( **B** and **D**). Binding to the promoter regions of *EGR1* and *EGR2* are shown and a negative control region located 2 kb upstream from the *EGR1* locus. Data are the average of 3 independent experiments. *p-value < 0.05.

**Figure 5.**
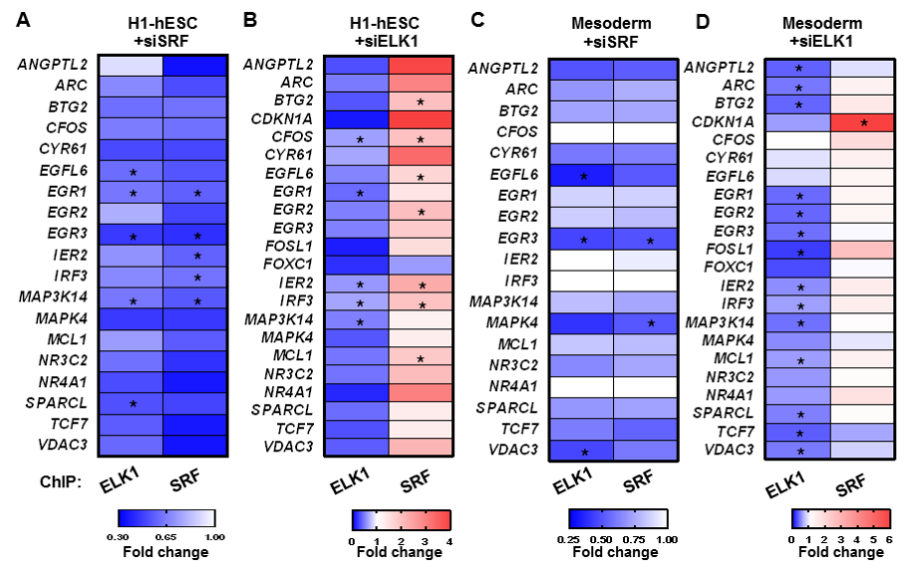
Inter-relationship between ELK1 and SRF binding to chromatin across co-bound loci. ( **A**– **D**) Heatmaps of chromatin immunoprecipitation-quantitative PCR showing fold change in ELK1 and SRF binding after treatment of either H1-hESCs with siSRF ( **A**) and siELK1 ( **B**) or mesoderm cells after treatment of either H1-hESCs with siSRF ( **C**) and siELK1 ( **D**). Data are the average of three independent experiments. *p-value <0.05.

To determine possible functional interactions between ELK1 and its known cofactor, SRF, we first identified a set of genes which are located close to potential regulatory regions that are co-bound by ELK1 and SRF. Next, we treated H1-hESCs with either siSRF or siELK1 and then either maintained the hESCs in their pluripotent state or differentiated them to mesoderm cells. First we analysed SRF and ELK1 binding to chromatin and performed ChIP-quantitative PCR (qPCR) on regions which were bound by both ELK1 and SRF. Binding of both factors was specifically detectable on the known target genes *EGR1* and *EGR2* ( Figure 4). Generally, we saw a decrease in SRF binding and a concomitant decrease in ELK1 binding in the same regions following SRF depletion ( Figure 4A, C and Figure 5A, C). This pattern was detected in 19/19 of the regions tested H1-hESC ( Figure 5A) and 18/22 of the regions tested in mesoderm ( Figure 5C). This is consistent with the existing models, whereby SRF acts as a platform to aid ELK1 recruitment to chromatin ( Gille *et al*., 1995; Janknecht & Nordheim, 1992; Latinkić *et al*.,1996; Treisman *et al*., 1992). Conversely, when we depleted ELK1 and performed ChIP-qPCR on regions which were bound by both ELK1 and SRF, we saw the expected decrease in ELK1 binding but a general increase in SRF binding in both H1-hESCs and mesoderm cells ( Figure 5B, D). This pattern was detected in 16/19 of the regions tested H1-hESC) ( Figure 5B) and 16 of the 22 regions tested in mesoderm ( Figure 5D). These results confirm a widespread role for SRF in stabilising ELK1 occupancy on chromatin but suggest an unexpected role for ELK1 in apparently reducing SRF occupancy on chromatin. Raw Fluidigm data are available on figshare ( Prise, 2019d).

**Figure 6.**
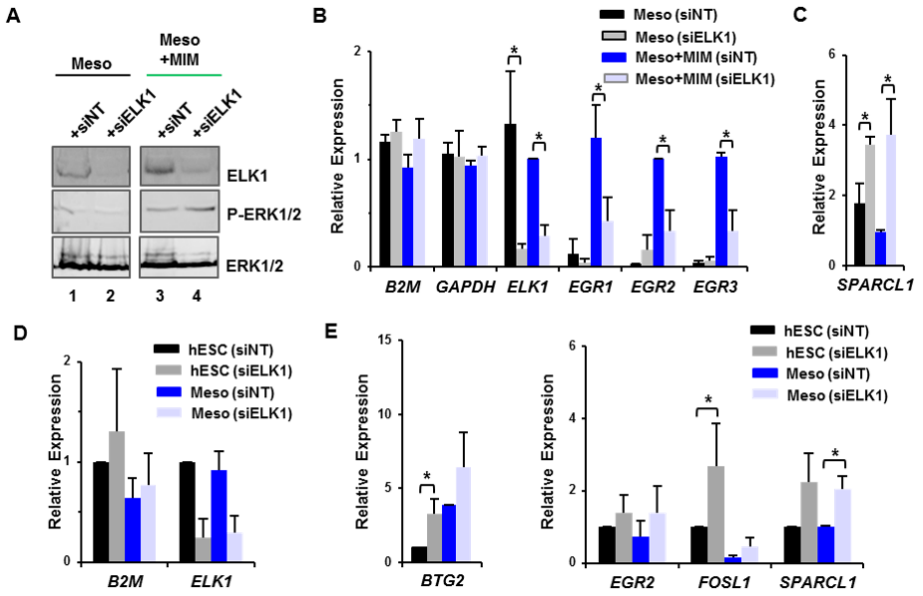
ELK1 is functions as an activator or a repressor at different target genes. ( **A**) Western blot showing ELK1, phospho-ERK1/2 (P-ERK) and ERK2 expression in MIM-differentiated mesoderm cells treated with non-targeting (NT) siRNA or siRNAs targeting ELK1 and either starved of MIM or grown with stimulation by MIM addition for 15 mins. ( **B** and **C**) Reverse transcription-quantitative PCR (RT-qPCR) showing the expression of control and ERK pathway activated genes (EGR1-3) ( **B**) and an exemplar novel ELK1-SRF co-bound bound gene ( **C**) in mesoderm cells treated with siELK1 and stimulated with MIM for 15 mins following serum starvation for 24 hrs. Data are normalised to siNT treated cells (taken as 1) and are the average of 3 independent experiments. ( **D** and **E**) RT-qPCR showing the expression of the indicated control genes ( **D**) or genes co-bound by ELK1 and SRF ( **E**) following treatment with non-targeting (NT) siRNA or siRNA directed against ELK1. Data are shown for H1-hESCs and differentiated mesoderm cells grown under steady state conditions. Data are normalised to levels in H1-hESCs in the presence of siNT (taken as 1) and are the average of three independent experiments. *p-value <0.05.

To investigate whether these changes in transcription factor binding correlated with gene expression, we again performed a knockdown of ELK1 in H1-hESCs or during mesoderm differentiation and tested the expression of a number of target genes. First, we assessed whether we could detect the known activating role of ELK1 at the immediate-early genes *EGR1*, *EGR2* and *EGR3* in these cells. As these genes are inducibly activated by ERK pathway signalling, we activated the ERK pathway by culturing mesoderm cells (derived from H1-hESCs) in DMEM/F12 media followed by serum starvation for 24 hrs and then switched the media to MIM for 15 mins. This treatment caused increased levels of active, phosphorylated ERK ( Figure 6A) and the activation of *EGR1*, *EGR2* and *EGR3* expression ( Figure 6B). ELK1 was efficiently depleted by siRNA treatment ( Figure 6A) and this depletion caused a significant decrease in the expression of *EGR1*, *EGR2* and *EGR3* under stimulating conditions ( Figure 6B). Having established the known activating role of ELK1 in our system, we switched to investigating whether ELK1 plays an activating role at different genes which are not expected to be activated by the ERK pathway as exemplified by *SPARCL1* ( Figure 6C). In both H1-hESC and mesoderm cells grown under steady state levels, ELK1 depletion caused an increase rather than a decrease in the expression of a panel of its target genes bound by the ELK1-SRF complex ( Figure 6D, E). This indicates that ELK1 is necessary for the repression rather than the activation of these target genes. ELK1 is also responsible for EGR2 repression in basal and steady state levels ( Figure 6B) but switches to an activating role following acute stimulation ( Figure 6E) which is consistent with previous data showing that it can both activate and repress transcription ( Marais *et al*., 1993; Yang *et al*., 2001; Lee *et al*., 2010).

### ELK1 status does not affect the response to changes in actin dynamics

A plausible model is that SRF is an activator at these loci, and ELK1 would then act in a repressive manner to keep SRF activity in check. This model was previously proposed for the role of ELK1 in opposing the recruitment of the MRTF family co-activator myocardin in smooth muscle cells ( Wang *et al*., 2004). Indeed, ELK1 and MRTFs occupy the same binding surface on SRF, meaning that binding is mutually antagonistic ( Zaromytidou *et al*., 2006). If ELK1 was opposing the actions of MRTF family members, depletion of ELK1 would be predicted to hypersensitise target gene expression to activators of this pathway. To address this possibility, we tested the effect of ELK1 depletion in the context of cytochalasin D stimulation, which acts through inhibiting actin polymerisation and has been shown to activate SRF-target genes through the MRTFs ( Esnault *et al*., 2014; Posern & Treisman, 2006).

**Figure 7.**
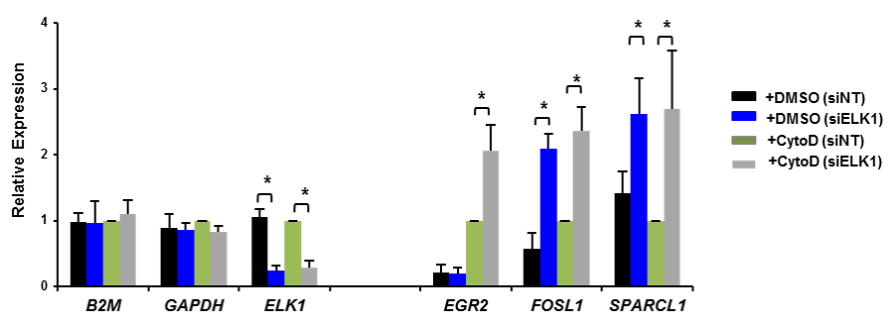
ELK1 depletion does not sensitise target genes to MRTF pathway activation. Reverse transcription-quantitative PCR showing the expression of the indicated control genes (left) or genes co-bound by ELK1 and SRF (right) following treatment with non-targeting (NT) siRNA or siRNA directed against ELK1. Data are shown for mesoderm cells treated with DMSO or cytochalasin D (cyto D). Data are the average of three independent experiments. *p-value <0.05.

 We stimulated serum-starved mesoderm cells with cytochalasin D, concurrently with ELK1 knockdown, and tested the expression of four target genes for the ELK1-SRF complex, *EGR2*, *CDNKN1A*, *FOSL1* and *SPARCL1*. Neither *FOSL1* nor *SPARCL1* were responsive to cytochalasin D treatment under serum-starved conditions, suggesting a lack of involvement of MRTFs ( Figure 7). As observed previously, we saw an increase in the expression of both genes upon ELK1 knockdown in mesoderm cells under both normal culture conditions and also when treated with cytochalasin D ( Figure 7). However, these genes did not become responsive to MRTF pathway activation when the putative MRTF-binding inhibition via ELK1 was removed by ELK1 depletion ( Figure 7). In contrast, *EGR2*, a gene usually activated by ELK1, is activated by cytochalasin D and this effect is potentiated by depletion of ELK1 ( Figure 7). Thus, ELK1 appears to interact differently with the MRTF pathway at different loci, but in regions where it acts as a repressor such as the *SPARCL* locus, it does so without influencing the response to this pathway. This suggests that binding by another co-activator or the intrinsic activity of SRF alone may be responsible for the increased gene activation we observe in the absence of ELK1.

## Discussion

**Figure 8.**
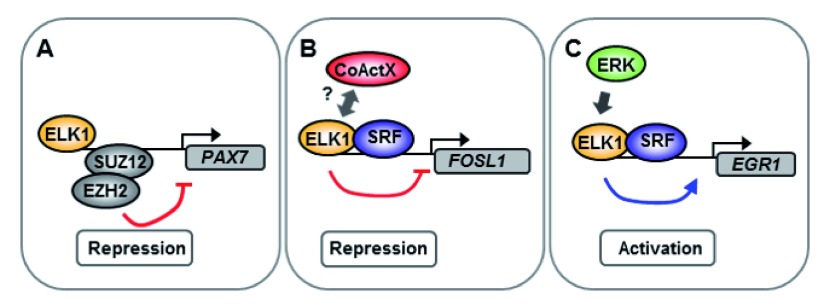
Model for the role of ELK1 at different genomic loci. ( **A**) ELK1 binds to the same genomic regions as SUZ12 and EZH2 of the PRC2 complex. In this scenario, repression is mediated at genes like *PAX7* by the PRC2 complex but not ELK1. ( **B**) ELK1 binds to regions alongside its partner protein SRF. In this scenario, repression is mediated at genes like *FOSL1* by ELK1, potentially by competing for another co-activator protein. ( **C**) ELK1 binds to regions alongside its partner protein SRF and is activated by the ERK pathway. Here ELK1 acts as a canonical transcriptional activator protein at genes like *EGR1.*

Previous work studying the role of ERK signalling in H1-hESC led to a focus on ELK1 as a potential regulator of pluripotency ( Göke *et al*., 2013). In this context, ELK1 was proposed to act in combination with the PRC2 complex to repress the expression of genes involved in hESC differentiation. In line with this previous analysis on promoter proximal events, we demonstrated that ELK1 binding occupies two distinct modules throughout the genome, one enriched for the repressive PRC2 complex and one enriched for the presence of active histone marks and binding of the known ELK1 partner protein SRF. However, we were unable to demonstrate a repressive role for ELK1 through these elements indicating that ELK1 does not act in the context of PRC2-mediated repression in hESCs. Although we have surveyed a panel of genes, it remains possible that other genes may be controlled through this complex and/or the repressive function is only revealed under particular conditions. Based on our analysis though, the widespread binding of ELK1 and the PRC2 complex appears to be coincidental rather than functionally linked ( Figure 8A).

This led us to question whether ELK1 acts merely as a transcriptional activator rather than a repressor in hESCs, consistent with its known role as a mediator of ERK pathway-mediated gene activation ( Gille *et al*., 1995; Janknecht *et al*., 1993; Marais *et al*., 1993). We confirmed that ELK1 acts as an activator of canonical target genes for the ELK1-SRF complex such as *EGR1* ( Figure 8C). However, we found that ELK1 loss broadly resulted in increased SRF ChIP signal at a panel of target genes for this complex, suggesting that it might act as a repressor in this context, perhaps by destabilising SRF binding to DNA. Indeed, we showed that ELK1 acts in a repressive manner at a subset of target genes for this complex, as exemplified by *FOSL1* ( Figure 8B). However, both ELK1 and SRF binding is detectable at this and other loci by ChIP-seq analysis, making it unlikely that ELK1 destabilises SRF binding. Moreover, previous studies have shown that ELK1 can stabilise SRF binding to DNA rather than inhibiting its binding ( Ling *et al*., 1998). Others have previously shown that depletion of ELK1 along with other TCF subfamily proteins, results in increased ChIP signal for SRF at co-bound genes in murine cells, although they provided no molecular explanation for this phenomenon ( Gualdrini *et al*., 2016). Instead, an alternative technical explanation might be increased epitope exposure on SRF after ELK1 knockdown, either through loss of steric hindrance or due to conformational changes in the DNA-bound SRF.

In theory, loss of ELK1 binding to SRF might reveal a binding surface for another co-activator protein as well as potentially allowing access to antibodies used in the ChIP procedure. Such a scenario would be consistent with the previously defined role for ELK1 in opposing binding by the MRTF family of co-activator proteins ( Wang *et al*., 2004; Zaromytidou *et al*., 2006). However, loss of ELK1 did not generally make its target genes more responsive to MRTF pathway activation. It remains possible that basal MRTF signalling may be operative in these cells and that ELK1 presecence opposes this and future additional studies are needed to fully rule out a role for MRTFs. Whilst our current data do not support a role for MRTFs as an important co-activator in this context, it may be that an as-yet-unknown factor may play a role in SRF-mediated gene activation. Future studies are needed to address this possibility. Alternatively, ELK1 may itself impart repressive properties on an SRF-bound regulatory region, such as through recruitment of the SIN3A complex ( Yang *et al*., 2001) or through SUMO-mediated recruitment of histone deacetylases ( Yang & Sharrocks, 2004) as previously shown in other cell types. However, it is unclear why ELK1 should be repressive at only a subset of its binding regions and activating at others. Finally, it remains possible that ELK1 may repress its targets through an indirect mechanism, although it appears more likely that it would act directly through its binding to the regulatory regions of its target genes.

In summary, our work has identified a role for ELK1 in acting in its traditional role as a transcriptional activator downstream from the ERK pathway in hESCs. In addition, it also plays a repressive role in hESCs through SRF-bound regulatory regions. However, we were unable to find evidence to support a role in transcriptional repression in conjunction with the PRC2 complex as previously proposed ( Göke *et al*., 2013). ELK1 therefore acts as dichotomous transcriptional regulator in hESCs, through imparting both activating and repressive activities to SRF-bound target genes.

## Methods

### Cell Culture

H1-hESC cells (Wicell) were routinely cultured in mTeSR1™ (StemCell Technologies). Plates were coated with Matrigel (Corning) at 37°C for 1 hour before passage. To passage the cells, the cells were coated with a thin layer of ReLeSR™ (StemCell Technologies) and incubated at 37°C for 5 minutes.

For shELK1 and RA treatment, conditioned H1 media was used, containing DMEM/F12 (Invitrogen), 20% (v/v) knockout serum replacement (Thermo Fisher Scientific), 1 mM L-glutamine (Gibco), 1% (v/v) nonessential amino acids (Gibco), 0.1 mM 2-mercaptoethanol (Gibco), and 4 ng/ml basic fibroblast growth factor (Invitrogen). This media was conditioned with CF1 mouse fibroblasts (MTI-GlobalStem) for 24 hr prior to adding to the H1-hESCs. Media was then vacuum filtered (0.22 µM), and an additional 8 ng/ml of basic fibroblast growth factor (Invitrogen) was supplemented to conditioned medium before usage. To passage the cells, the cells were coated with a thin coat of dispase (StemCell Technologies) and incubated at 37°C for 5 minutes.

For differentiating hESCs to mesoderm, cells were first treated with 10 µM Y-27632 (ROCK inhibitor) for 1 hour before passage and dissociated with TrypLE™ Express (StemCell Technologies). Cells were initially seeded at a density of 5×10 ^5^ per cm ^2^ in mTeSR1™ supplemented with 10 µM Y-27632. After 24 h, cells were grown in STEMdiff™ mesoderm Induction medium (StemCell Technologies) for an additional 3 days.

To activate the ERK signalling pathway, H1-hESCs were first allowed to differentiate into mesoderm cells, maintained in DMEM/F12 media lacking serum for 24 h and then switched the media to MIM for 15 mins for analysing ERK activation and 60 mins for studying gene expression. DMEM/F12 media was used as a control for MIM stimulation.

For retinoic acid (RA) treatment, cells were dissociated with TrypLE™ Express and seeded at a density of 5×10 ^5^ per cm ^2^ in mTeSR1™ supplemented with 10 µM Y-27632. After 24 h, cells were grown in in mTeSR1™ supplemented with 5 µM RA (Sigma) for up to 96 h. DMSO was used at a final concentration of 1:10,000 as a control for RA treatment.

For cytochalasin D stimulation,  H1-hESCs were first allowed to differentiate into mesoderm cells, maintained in DMEM/F12 media lacking serum for 24 h and then switched to DMEM/F12 containing 2 μM  cytochalasin D for 60 minutes to study gene expression. DMEM/F12 media containing DMSO at a final concentration of 1:10,000 was used as a control for cytochalasin D stimulation.

### shRNA and siRNA treatment regimes

For shRNA treatment, cells were first treated with ROCK inhibitor and dissociated with TrypLE™ Express. Next, 5×10 ^5^ cells were treated with 7.5 µl of TransIT®-LT1 (Mirus) plus 2.5 µg of shRNA plasmid prepared in 250 µl of OptiMEM. shELK1 plasmid was a pSuper-derived plasmid containing the shRNA hairpin for ELK1: 5’-GCCAGAAGTTCGTCTACAA-3’ ( Göke *et al*., 2013). An empty pSuper plasmid was used as a control for shELK1 treatments.

For siRNA transfection cells were prepared as above and after dissociation seeded at a concentration of 5×10 ^5 ^per cm ^2 ^in mTeSR1™ supplemented with 10 µM Y-27632. Each 5×10 ^5 ^cell sample was treated with 7.5 µl Lipofectamine RNAiMAX reagent (Thermo Fisher Scientific) and 2.5 µl of siRNA (20 µM stock concentration), prepared in 150 µl Opti-MEM (Gibco). A non-targeting siRNA (siNT) was used as a control for siELK1 and siSRF treaments.

### ChIP assays

Cells were incubated at room temperature with 1% (v/v) formaldehyde (Sigma), for 10 minutes (3×10 ^6^ cells were seeded per immunoprecipitation (IP)). The crosslinking reaction was then quenched with 0.125 M glycine for 5 minutes. Cells were washed with ice cold 1x PBS. Next, 3×10 ^6^ cells were harvested in FA cell lysis buffer (10 mM Tris-HCl, pH 8.0, 0.25% (v/v) Triton-X100, 10 mM EDTA, 0.1M NaCl) rotated for 10 minutes at 4°C, the nuclei pelleted at 13.1 krpm at 4°C for 5 minutes and the supernatant discarded. Cells were resuspended in FA Cell Lysis Buffer, rotated for 10 minutes at 4°C, pelleted at 13.1 krpm at 4°C for 5 minutes and the supernatant discarded. Nuclei were then resuspended in 1% SDS solution (50 mM HEPES-KOH, pH 7.5, 150 mM NaCl, 2 mM EDTA, 1% (v/v) Triton-X100, 0.1% (v/v) Na-DOC, 1% (w/v) SDS), rotated for 10 minutes at 4°C and the chromatin pelleted at 13.1 krpm at 4°C for 5 minutes and the supernatant discarded. Chromatin was then suspended in 0.1% SDS solution (50 mM HEPES-KOH, pH 7.5, 150 mM NaCl, 2 mM EDTA, 1% (v/v) Triton-X100, 0.1% (v/v) Na-DOC, 0.1% (w/v) SDS), rotated for 10 minutes at 4°C, pelleted at 13.1 krpm at 4°C for 5 minutes and the supernatant discarded. Chromatin was then resuspended in 0.1% SDS solution and sonicated to produce chromatin fragments of 100–500 bp.

For 3×10 ^6 ^cells, 12.5 µl of Dynabeads® Protein G (Thermo Fisher Scientific) and 1.25 µg of antibodies were conjugated at room temperature for 2 hours, after which, conjugated beads were washed with 0.1% SDS buffer. The lysate was then rotated with the conjugated beads overnight at 4°C. The next day, beads were washed sequentially with 0.1% SDS solution high salt wash (50 mM HEPES-KOH, pH 7.5, 500 mM NaCl, 2 mM EDTA, 1% (v/v) Triton-X100, 0.1% (v/v) Na-DOC, 0.1% (w/v) SDS), NP40/LiCl wash (10 mM Tris-HCl, pH8.0, 250 mM LiCl, 1 mM EDTA, 0.5% (v/v) NP-40, 0.1% (w/v) Na-DOC)and TE (1 0M Tris-HCl pH 8.0, 1 mM EDTA). The beads were then resuspended in ChIP elution buffer (50 mM Tris-HCl, pH 7.4, 10 M EDTA, 1% (w/v) SDS) and shaken at 69°C at 1000 rpm for 1 hour. This supernatant was then transferred to a new tube, treated with 1:50 Proteinase K (20 mg/ml) (Roche) and shaken at 55°C at 600 rpm for 1 hour. For siELK1 and MIM treatment, DNA was then further purified using the QIAquick PCR purification kit (Qiagen). For shELK1 and RA treatment, the eluted DNA was mixed with an equal volume of phenol-chloroform (Thermo Fisher Scientific). The aqueous layer was isolated following centrifugation, for 10 minutes at 13.1 krpm at 4°C, and mixed 1:1 with isopropanol and frozen at -80°C for 30 minutes. The solution was then spun at 13.1 krpm at 4°C for 20 minutes and the supernatant discarded. The pellet was then washed with 70% ethanol and spun two more times. It was then air-dried for 24 h and 100 µl of water was added.

A PCR reaction was then run with the following settings: 50°C for 30 min (only for RT-PCR), followed by 95°C for 20 min then [95°C for 20 s, 55°C for 30 s, 72°C for 30 s] for 40 cycles, melt curve 72-95°C. ChIP-qPCR samples in Figure 3 and RT-qPCR in Figure 4 were analysed with the BioMark HD System (Fludigim), used as per the manufacturer’s instructions. The 14-cycle Specific Target Amplification was used for pre-amplification of the ChIP product and Exonuclease I treatment was used to remove unincorporated primers. BioMark Data Analysis (Fluidigm) was used for data analysis. PCR primers are shown in Extended data, Supplementary Table S1 ( Sharrocks, 2019). 

### RT-qPCR assays

For MIM stimulation and siELK1 experiments, RNA was purified with an RNeasy Kit (Qiagen) using the manufacturer’s protocol. For RA and shELK1 experiments, cells were collected into 350 µl of RNAzol (Sigma) homogenised with a Gilson pipette, and spun at 13.1 krpm at 4°C for 20 minutes The aqueous layer was then mixed 1:1 with isopropanol and RNA precipitated and dried as described for ChIP-isolated DNA above. cDNA was then prepared using SuperScript2 (Thermo Fisher Scientific) according to the manufacturer’s instructions (PCR was then carried out using Power SybrGreen (Thermo Fisher Scientific) with an annealing temperature of 55°C, according to the manufacturer’s instructions. Data was collected with the ViiA 7 (Thermo Fisher Scientific) and analysed with Viia7 V1.2 software (Thermo Fisher Scientific). The PCR primers are shown in Extended data, Supplementary Table S1 ( Sharrocks, 2019).

### Western blotting

For Western blot analysis, cells were harvested in RIPA buffer by scraping on ice. Cell lysates were then centrifuged at 13.1 krpm at 4°C for 2 minutes. The supernatant was then measured using a Bradford Protein Assay. Next, 1 µl of sample was added to 1 ml of Coomassie Brilliant Blue (ThermoFisher Scientific) and measured against BSA standards ranging from 0.2 mg/ml to 2 mg/ml. Approximately 20 µg of protein was used for each well. 1x SDS loading buffer was added to the lysate, which was then boiled at 99°C for 10 minutes. Proteins were resolved on the 12% gel in 1x SDS running buffer, and transferred to a nitrocellulose membrane using transfer buffer. Finally, cells were incubated with primary and secondary antibodies ( Extended data, Supplementary Table S2 ( Sharrocks, 2019)), diluted 1:500–2000 and 1:10000, respectively, in Licor Odyssey Buffer and imaged on the Odyssey Imaging System (Licor biosciences).

### Bioinformatics analysis

For ChIP-seq analysis from published datasets from human H1-hESCs (in Extended data, Supplementary Table S3 ( Sharrocks, 2019)), reads were mapped to the genome using Bowtie2 (v2.2.9) with default settings ( Langmead & Salzberg, 2012). Bowtie2 output was then sorted, compressed and unaligned reads removed using samtools (v0.1.18), using the settings -Shu -F4, which removed unmapped reads ( Li *et al*., 2009). Finally, peaks were called with MACS2, using the default settings ( Zhang *et al*., 2008). To identify intersecting peaks from two datasets, after MACS2 peak calling, narrowPeak files were intersected with the intersectBed tool in bedtools (v2.21)( Quinlan & Hall, 2010), using the -f 0.1 and -r settings, creating a reciprocal overlap of 10%.

To create tag density graphs, the mapped, sorted and compressed ChIP-seq files were converted to BED files using the bamtobed tool in bedtools. BED files were converted to tag directories using the makeTagDirectory.pl tool in HOMER (v4.8.3) ( Heinz *et al*., 2010). Finally, ChIP-peaks were annotated using annotatePeaks.pl tool in HOMER, using the settings -size -2500, 2500 -hist 25 -norm 0, which created a tag density profile with a 25 bp bin, averaged to tag count in all tag directories, 2500 bp on either side of the peak centre.

To associate peaks to potential target genes we used the nearest gene model in HOMER (with default settings; Heinz *et al*., 2010). Enriched functional or biological processes associated with these genes were identified from lists of gene ontology (GO) terms using DAVID v6.7 ( Huang *et al*., 2008; Huang *et al*., 2009).

### Statistical analysis

Pairwise student’s t-tests were performed in GraphPad v7. Statistical significance determined using the Holm-Sidak method, with significance set to p < 0.05. Hypergeometric p-values were calculated using the phyper function in R v3.4.1

## Data availability

### Underlying data

Raw data underlying the findings of this study are available from figshare. These include raw GO data ( Figure 1F, G: GO terms), RT-qPCR data ( Figure 2A: RT-qPCR data for RA stimulation; Figure 2B, D–F: RT-qPCR data plus shELK1; Figure 3A: RT-qPCR data for pluripotency genes in hESCs and mesoderm cells; Figure 3E: RT-qPCR data for pluripotency genes plus siELK1; Figure 3F: RT-qPCR data for differentiation factor genes plus siELK1; Figure 6D: RT-qPCR data in mesoderm and hESCs plus siELK1; Figure 6B and C: RT-qPCR data for *EGR1-3* plus/minus MIM stimulation; and Figure 7: RT-qPCR data with CytoD treatment), ChIP-qPCR data ( Figure 2D: ChIP-qPCR data for ELK1 plus siELK1; Figure 2E: ChIP-qPCR data for SUZ12 plus siELK1), Fluidigm data ( Figure 4 and Figure 5: ChIP-qPCR data plus siELK1 or siSRF), and uncropped western blots ( Figure 2C, Figure 3C, D and Figure 6).

DOIs: GO, https://doi.org/10.6084/m9.figshare.7657958 ( Prise, 2019a); RT-qPCR, https://doi.org/10.6084/m9.figshare.7667099 ( Prise, 2019b); ChIP-qPCR, https://doi.org/10.6084/m9.figshare.7657961 ( Prise, 2019c); Fluidigm, https://doi.org/10.6084/m9.figshare.7657931 ( Prise, 2019d); western blots, https://doi.org/10.6084/m9.figshare.7675682 ( Prise, 2019e).

### Extended data

Extended data are available from figshare.

Supplemental Table S1. Human H1-hESCs ChIP-seq data set accession numbers used in this study.

Supplemental Table S2. Antibodies used in the study.

Supplemental Table S3. Human H1-hESCs ChIP-seq data set accession numbers used in this study.

DOI: https://doi.org/10.6084/m9.figshare.7695617 ( Sharrocks, 2019).

Data are available under the terms of the Creative Commons Attribution 4.0 International license (CC-BY 4.0).
