# Supplementary material for: ELK1 has a dual activating and repressive role in human embryonic stem cells
Source: Wellcome Open Res. 2019 Jul 5;4:41. Originally published 2019 Mar 1. [Version 2] doi: 10.12688/wellcomeopenres.15091.2 (PMC6619381; doi:10.12688/wellcomeopenres.15091.2)
Supplement: Peer review history

# Peer review: Reviewer response for version 2

**DOI:** 10.21956/wellcomeopenres.16758.r35922

**Published:** 2019-07-08

**Authors:** Ron Prywes

**Affiliations:** 1 Department of Biological Sciences, Columbia University, New York, NY, 10027, USA

The authors have satisfactorily responded to my comments. While they have not ruled out a role for Elk1 repression through blocking MRTF-A/B binding to SRF, they have qualified their conclusions. (This still appears plausible for the Egr2 gene, which is activated by Cytochalasin D in Figure 7, and further activated by Elk1 knockdown.)  In general this is an interesting study showing Elk1 repression of target genes co-bound by SRF. The mechanism of Elk1 repression remains to be determined.

I confirm that I have read this submission and believe that I have an appropriate level of expertise to confirm that it is of an acceptable scientific standard.

# Peer review: Reviewer response for version 2

**DOI:** 10.21956/wellcomeopenres.16758.r35921

**Published:** 2019-07-08

**Authors:** Alexis Verger

**Affiliations:** 1 UMR 8576, Unité de Glycobiologie Structurale et Fonctionnelle (UGSF), CNRS, Université de Lille, Lille, France

The authors have addressed appropriately all my concerns.

I confirm that I have read this submission and believe that I have an appropriate level of expertise to confirm that it is of an acceptable scientific standard.

# Peer review: Reviewer response for version 1

**DOI:** 10.21956/wellcomeopenres.16464.r34990

**Published:** 2019-03-21

**Authors:** Alexis Verger

**Affiliations:** 1 UMR 8576, Unité de Glycobiologie Structurale et Fonctionnelle (UGSF), CNRS, Université de Lille, Lille, France

In this paper, the authors evaluate the dual activating and repressive role of the ETS transcription factor ELK-1 in human embryonic stem cells. The paper builds upon a previous study (Göke et al. Molecular Cell 2013 ^1^) in which ERK genome-wide chromatin interactions in human embryonic stem cells was characterized. In the present paper, the authors observed a significant overlap in ELK-1 and SUZ12 (PRC2 complex) binding regions but they don’t find any evidence that ELK-1 does have a role in mediating transcriptional repression through these regions. Interestingly they find that ELK-1 has a repressive role during mesoderm differentiation that appears not to be exerted through competition with MRTF co-activators of RhoA-mediated gene expression. The paper represents an interesting follow up of the earlier work and is written in a clear and logical fashion. The purpose of the study is well defined and technically sound. However some data and aspects of the manuscript are worth clarifying.

**Figure 1.** My understanding is that ELK-1 binding motif is only enriched in the subset of ELK-1 binding sites that are co-occupied by ERK2 whereas promoters bound by ELK-1 without ERK2 (and thus with SUZ12 and EZH2) do not have this ELK-1 binding motif, at least for a 500bp window ^1^. Is this also the case with your 2500bp window? If true, do the authors have any explanation of how ELK-1 is recruited?

**Figure 2A.** The RT-qPCR experiments after retinoic acid (RA) treatment appear somewhat highly variable especially for PAX7 and POUF3F2. Do the authors identify more than the six ELK-1+SUZ12 bound genes whose expression increased upon RA treatment? If yes, it should be worth to include them. Is the expression of ELK-1 affected by RA?

**Figure 3.** The role of ELK-1 during mesoderm expression should be discussed in more detail. The authors nicely show that ELK-1 depletion caused an increase in the expression of FOXC1 and HAND2 (Figure 3F) but in the meantime the Western-blot (Figure 3C) suggests that ELK-1 protein expression is induced during mesoderm differentiation. Is this possible that these genes are ELK-1/PRC2- mediated repression dependent?

**Figure 5.** FOXC1 (in relation with RT-qPCR Figure 3F) is included in the heatmaps of chromatin Ip but not HAND2. If the ChiP-qPCR has been done for HAND2, it could be nice to add it.

I confirm that I have read this submission and believe that I have an appropriate level of expertise to confirm that it is of an acceptable scientific standard, however I have significant reservations, as outlined above.

## References

[1] : Genome-wide kinase-chromatin interactions reveal the regulatory network of ERK signaling in human embryonic stem cells. *Mol Cell*.2013;50(6) : 10.1016/j.molcel.2013.04.030 844-55 10.1016/j.molcel.2013.04.030 23727019

# Peer review: Reviewer response for version 1

**DOI:** 10.21956/wellcomeopenres.16464.r34992

**Published:** 2019-03-12

**Authors:** Ron Prywes

**Affiliations:** 1 Department of Biological Sciences, Columbia University, New York, NY, 10027, USA

The authors nicely evaluate the role of Elk1 in regulating gene expression in human embryonic stem cells. Elk1 co-binds to DNA with SRF, but can also bind DNA without SRF. They don’t find evidence that the sites bound without SRF are functional here. Interestingly, they find that Elk1 can function as an activator or a repressor depending upon the exact gene binding site they examine.

 One conclusion that I feel is less well proven is that Elk1 repression is independent of its blocking another SRF co-factor MRTF-A and -B. Their evidence is that activation of MRTF-A/B by cytochalasin D is not stimulated after Elk1 knockdown. However, the activation of specific genes in these cells could be due to basal MRTF-A/B activation (or is the related myocardin gene expressed in these cells?). If so, perhaps there is something about these cells that limits cytochalasin activation of MRTF-A/B at these genes. A more thorough way to test this question is to knockdown the members of the MRTF family along with Elk1 knockdown to see whether the derepression is affected.

 Some more minor points:

1. In Figure 6, different cell treatments are used in 6B and 6D, E. The differences are not entirely clear. In 6B, there is a brief treatment of MIM media. What is done in the other figures? This is important as in 6B, Elk1 activates expression and in 6E it represses.
2. It’s concluded for Fig. 6, on page 6, that Elk1 represses specific genes. This implies that it is doing so directly by binding to them. What rules out an indirect effect? This should at least be discussed.
3. In Figure 2, the authors examine retinoic acid induction of the cells and conclude that Elk1 binding to PRC2 is not important. Later in the paper they use media to induce the cells to differentiate to mesodermal cells. Is it possible that Elk1/PRC2 target genes are important for this type of induction of these cells?
4. We previously found that Elk1 could act as a repressor of MRTF target genes in NIH3T3 cells particularly when grown continuously ^1^ and found a redundancy for immediate early activation of some genes by Elk1 and MRTF. Are the embryonic stem cells different than other cell types for Elk1 activity and does the growth state of the cells affect Elk1 activity?

I confirm that I have read this submission and believe that I have an appropriate level of expertise to confirm that it is of an acceptable scientific standard, however I have significant reservations, as outlined above.

## References

[1] : Activation and repression of cellular immediate early genes by serum response factor cofactors. *J Biol Chem*.2010;285(29) : 10.1074/jbc.M110.108878 22036-49 10.1074/jbc.M110.108878 20466732PMC2903344
